# Alleviative Effect of *Ruellia tuberosa* L. on Insulin Resistance and Abnormal Lipid Accumulation in TNF-*α*-Treated FL83B Mouse Hepatocytes

**DOI:** 10.1155/2021/9967910

**Published:** 2021-06-23

**Authors:** Hong-Jie Chen, Chih-Yuan Ko, Jian-Hua Xu, Yu-Chu Huang, James Swi-Bea Wu, Szu-Chuan Shen

**Affiliations:** ^1^Department of Nutrition, People's Hospital of Leshan, Leshan 614000, China; ^2^Department of Clinical Nutrition, The Second Affiliated Hospital of Fujian Medical University, Quanzhou 362000, China; ^3^Department of Respiratory and Critical Care Medicine, The Second Affiliated Hospital of Fujian Medical University, Quanzhou 362000, China; ^4^School of Public Health, Fujian Medical University, Fuzhou, Fujian 350122, China; ^5^Respiratory Medicine Center of Fujian Province, Quanzhou 362000, China; ^6^Department of Tumor Surgery, The Second Affiliated Hospital of Fujian Medical University, Quanzhou 362000, China; ^7^Graduate Program of Nutrition Science, National Taiwan Normal University, Taipei 11677, Taiwan; ^8^Graduate Institute of Food Science and Technology, National Taiwan University, Taipei 10672, Taiwan

## Abstract

Type 2 diabetes mellitus (T2DM) is a chronic metabolic disease, and most patients with T2DM develop nonalcoholic fatty liver disease (NAFLD). Both diseases are closely linked to insulin resistance (IR). Our previous studies demonstrated that *Ruellia tuberosa L*. (RTL) extract significantly enhanced glucose uptake in the skeletal muscles and ameliorated hyperglycemia and IR in T2DM rats. We proposed that RTL might be via enhancing hepatic antioxidant capacity. However, the potent RTL bioactivity remains unidentified. In this study, we investigated the effects of RTL on glucose uptake, IR, and lipid accumulation *in vitro* to mimic the T2DM accompanied by the NAFLD paradigm. FL83B mouse hepatocytes were treated with tumor necrosis factor-*α* (TNF-*α*) to induce IR, coincubated with oleic acid (OA) to induce lipid accumulation, and then, treated with RTL fractions, fractionated with n-hexane or ethyl acetate (EA), from column chromatography, and analyzed by thin-layer chromatography. Our results showed that the ethyl acetate fraction (EAf2) from RTL significantly increased glucose uptake and suppressed lipid accumulation in TNF-*α* plus OA-treated FL83B cells. Western blot analysis showed that EAf2 from RTL ameliorated IR by upregulating the expression of insulin-signaling-related proteins, including protein kinase B, glucose transporter-2, and peroxisome proliferator-activated receptor alpha in TNF-*α* plus OA-treated FL83B cells. The results of this study suggest that EAf2 from RTL may improve hepatic glucose uptake and alleviate lipid accumulation by ameliorating and suppressing the hepatic insulin signaling and lipogenesis pathways, respectively, in hepatocytes.

## 1. Introduction

Type 2 diabetes mellitus (T2DM) is a metabolic disorder characterized by chronic hyperglycemia, insufficient insulin secretion or action, or insulin resistance (IR). These conditions cause abnormalities in lipid, carbohydrate, protein, water, and electrolyte functions. Long-term hyperglycemic conditions increase both oxidative stress and inflammatory responses, leading to an increased risk of chronic inflammatory systemic diseases and their respective complications [1–3].

Interestingly, approximately 70% of patients with T2DM present with nonalcoholic fatty liver disease (NAFLD), which is a bidirectional relationship [4, 5]. The main pathological feature of NAFLD is lipid overaccumulation in liver cells, and it is associated mainly with obesity and dyslipidemia. There is a strong relationship between IR and hepatocyte abnormal lipid accumulation. Normally, insulin promotes assimilation, stimulates glycogen synthesis in liver and muscle cells, and synthesizes and stores fat as adipocytes. If IR occurs, the lipolysis rate of visceral fat cells increases, which leads to an increased content of free fatty acids (FFAs) in the blood. When glycerol and fatty acids enter the liver, this results in increased lipogenesis and excess lipid accumulation in liver cells, eventually causing fatty liver disease. Taken together, the pathophysiology of NAFLD and T2DM is related to IR, and they are mutually vicious interrelated risk factors [4, 5], although their pathological mechanisms have not been fully elucidated. Current T2DM- or NAFLD-related studies follow combined approaches, and these researchers' paradigm is more in line with the actual condition of such patients.

Oleic acid (OA) is a monounsaturated fatty acid that is naturally found widely in animals and plants and is one of the most abundant fatty acids in human fat tissue. In fact, OA has been demonstrated to induce hepatic cell steatosis in established *in vitro* steatosis models [6, 7].

Our previous study has demonstrated that *Ruellia tuberosa* L. (RTL) aqueous or ethanolic extracts significantly improved glucose uptake in C2C12 myoblasts, alleviated tumor necrosis factor-*α*- (TNF-*α*-) induced IR in skeletal muscles, and ameliorated hyperglycemia and IR indices in high-fat-diet-fed plus streptozotocin-induced T2DM rats [8]. Additionally, we demonstrated that RTL ameliorated abnormal hepatic detoxification by enhancing hepatic antioxidant capacity [9]. However, the potent bioactivities of RTL and mechanism on improving NAFLD remain uncertain. Hence, in the present study, we aimed to utilize the TNF-*α*-induced IR combined with the OA-induced steatosis paradigm to identify the active ingredients of RTL extracts through thin-layer chromatography (TLC), to improve glucose intake and inhibit lipogenesis, and to clarify the underlying mechanisms of metabolism in FL83B cells.

## 2. Materials and Methods

### 2.1. Preparation of RTL Extracts

**Figure 1:**
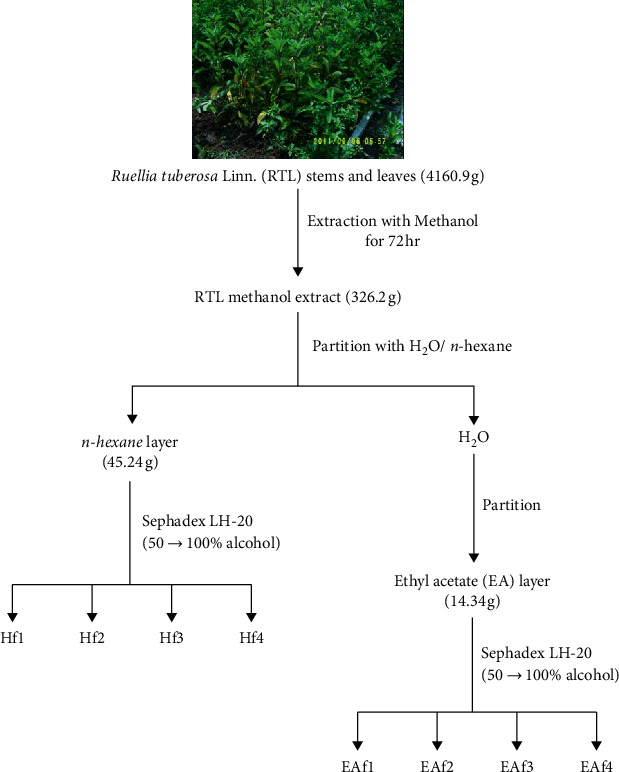
The flow chart for fractionation of *Ruellia tuberosa* Linn. extraction.

RTL was purchased in July 2017 from Wanhua, Taipei County, Taiwan, identified by Prof. Wei-Jan Huang in the College of Pharmacy, Taipei Medical University. A voucher specimen (TMU27449) was deposited in the herbarium of College of Pharmacy, Taipei Medical University. The extraction protocol was based on our previous methods, with slight modifications [8]. RTL stems and leaves together were freeze-dried into a powder (4160.9 g), subsequently extracted with 6 mL distilled water or methanol (1 : 6, w/v) at 4°C for 72 h, and filtered using a cheese cloth. The filtrate was filtered twice through Whatman No. 1 filter paper and centrifuged at 7,000 ×g for 20 min. The supernatant was vacuum-concentrated using a rotary evaporator below 40°C. The concentrated methanol extract was dissolved in 400 mL water and subsequently extracted with an equal volume of hexane and ethyl acetate (EA) solvent, and the n-hexane and EA layers were divided through column chromatography using a Sephadex LH-20 column with 200 mL of 50–100% alcohol sequentially. The different fractions were then collected from the n-hexane layer (Hf1, Hf2, Hf3, and Hf4) and the EA layer (EAf1, EAf2, EAf3, and EAf4), as shown in Figure 1.

### 2.2. TLC

The supernatants were placed onto a silica gel precoated plate (Kieselgel 60 F254, 0.20 mm, Merck, Darmstadt, Germany). The TLC plates were added with a solvent mixture of dichloromethane: methanol: H_2_O: acetic acid (10 : 1:0.1 : 0.2, v/v), followed by immersion into 10% sulfuric acid, and then, the mixture was heated. Using the color distribution state of TLC, similar effluents were collected, and the solvent was drained through a rotary evaporator. Different concentrates were freeze-dried into a powder and kept at −80°C until use.

### 2.3. Cell Culture

The experiments were performed on FL83B mouse hepatocytes (ATCC, Rockville, MD, USA) incubated in an F12K medium containing 10% fetal bovine serum (Invitrogen Corporation, Camarillo, CA, USA) in 10 cm Petri dishes at 37°C and 5% CO_2_. The experiments were performed on cells that were 80–90% confluent.

### 2.4. TNF-*α* Induction of IR in FL83B Cells

The IR-induced paradigm was described as the previous method with slight modifications [10]. FL83B cells were seeded in 10 cm dishes and incubated at 37°C for 48 h to reach 80% confluence. The serum-free medium containing recombinant mouse TNF-*α* and different RTL fractions (25 *μ*g/mL) was incubated for 2 h at 37°C to induce IR.

### 2.5. TNF-*α* Induction of IR with OA-Induced Lipid Accumulation in FL83B Cells

FL83B cells (5 × 10^5^) were seeded in six-well plates and incubated in a serum-free medium containing 20 ng/mL TNF-*α*, 1 mM OA, and 2% bovine serum albumin for 24 h. The cells were then transferred to a serum-free medium containing 25 *μ*g/mL of the samples and 100 nM of insulin, followed by incubation at 37°C.

Lipid accumulation was measured by using Oil Red O staining. Cells were placed on a plate, washed in PBS, and then, fixed in 10% formalin for 1 h. PBS was washed twice, and 0.5 g Oil Red O stain was added for 15 min. The stained cells were washed in distilled water, subsequently washed in 1ml isopropanol. Taking 100 *μ*l to a plate, the absorbance was measured at 492 nm. The value represented the total fat content in the cell.

### 2.6. Uptake of Fluorescent 2-[N-(7-nitrobenz-2-oxa-1,3-diazol-4-yl) amino]-2-deoxy-d-glucose (2-NBDG) in FL83B Cells

The FL83B cells were seeded in 10 cm dishes and then incubated at 37°C for 48 h to achieve 80% confluence. The serum-free medium containing 20 ng/mL recombinant mouse TNF-*α* was added before incubating for 2 h to induce IR. The cells were then transferred to another F12K medium containing 5 mM glucose, without (basal) or with 200 *μ*M insulin and 10 *μ*L different RTL fractions and incubated for 30 min at 37°C. An assay of glucose uptake was then performed as described previously [10].The fluorescence intensity of the cell suspension was evaluated by flow cytometry (FACScan, Becton Dickinson, Bellport, NY, USA) at an excitation wavelength of 488 nm and an emission wavelength of 542 nm. Fluorescence intensity reflected the cellular uptake of 2-NBDG.

### 2.7. Western Blot Analysis

The protein extraction methods used in this study were adopted from our previous protocol [11]. The protein concentration in the cell extract was determined using a Bio-Rad protein assay dye reagent (Richmond, VA, USA). Aliquots of the supernatant containing 50 *μ*g protein were separated through standard SDS-PAGE and electrophoretically transferred to polyvinylidene difluoride membranes. The membranes were incubated with anti-insulin receptor *β* (IR*β*; 1 : 1000; Cell Signaling Technology, Beverly, MA, USA), anti-PI3-kinase p85 (1 : 1000; Cell Signaling Technology), anti-phospho-Akt (Ser473) (1 : 1000; Cell Signaling Technology), anti-Akt (1 : 500; Cell Signaling Technology, Danvers, MA, USA), antiglucose transporter (GLUT)2 (1 : 500; Millipore, Billerica, MA, USA), anti-PPAR*α* (1 : 1000; GeneTex, Irvine, CA, USA), or anti *β*-actin (1 : 4000; GeneTex) antibodies at 4°C overnight. The membranes were incubated with anti-mouse IgG or anti-rabbit IgG secondary antibodies and washed thrice for 5 min each time. Protein band images were detected and captured using the UVP Biospectrum image system (Level, Cambridge, UK). Finally, all relevant protein expressions were normalized with *β*-actin.

### 2.8. Statistical Analyses

Values are presented as the mean ± standard deviation using SPSS version 22.0 (SPSS Inc., Chicago, IL, USA) analysis by one-way analysis of variance (ANOVA) and Duncan's new multiple-range tests. *p* < 0.05 was considered statistically significant.

## 3. Results

### 3.1. RTL Fractions Collection and Analysis by TLC

The different RTL fractions from the n-hexane layer (Hf1, Hf2, Hf3, and Hf4) and the EA layer (EAf1, EAf2, EAf3, and EAf4) were collected through TLC analysis (Supplementary Figure 1).

### 3.2. Effect of Different RTL Fractions on Glucose Uptake in FL83B Mouse Hepatocytes

**Figure 2:**
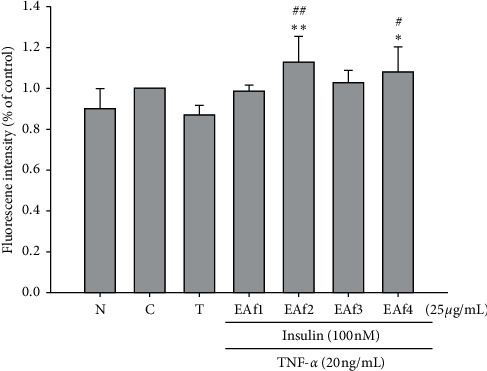
Effects of fractions from RTL extracts on glucose uptake in insulin-resistant FL83B cells. N cells incubated with the F12K medium group. C cells incubated with the F12K medium containing the 100 nM insulin group. T: TNF-*α* induced for the insulin resistance group. ^*∗*^*p* < 0.05 and ^*∗∗*^*p* < 0.01 vs. control group; ^#^*p* < 0.05 and ^##^*p* < 0.01 vs. TNF-*α* group.

An evaluation of the 2-NBDG uptake was performed to assess the improvement of glucose uptake in FL83B cells. The EAf4 fraction improved glucose uptake of FL83B mouse hepatocytes compared with the control group (*p*=0.030) or compared with the TNF-*α*-induced IR group (*p*=0.013). In addition, the fluorescence level of the EAf2 fraction group was 1.3 times greater than that of the TNF-*α*-induced IR group (*p*=0.014) (Figure 2).

### 3.3. Effect of EAf2 Fraction on TNF-*α* plus OA-Induced IR and Lipid Accumulation in FL83B Cells

**Figure 3:**
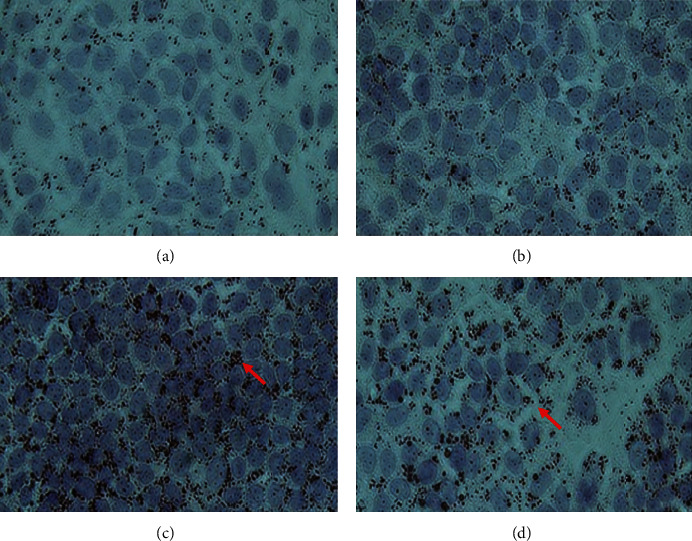
Lipid accumulation in FL83B cells by oil red O staining. (a) C: cells incubated with the F12K medium group. (b) T: TNF-*α* induced for the insulin resistance group. (c) OA: oleic acid induced for the lipid accumulation group. (d) TA: TNF-*α* and oleic acid induced insulin resistance for thefatty liver cells group.

**Figure 4:**
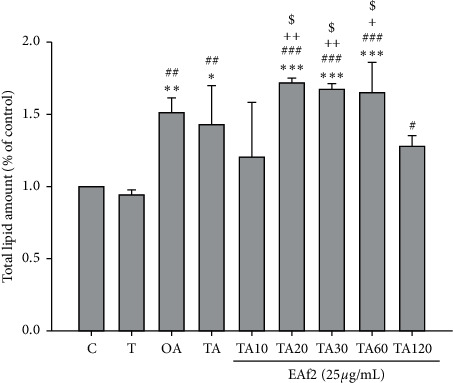
Effects of ethyl acetate fraction 2 (EAf2) on lipid accumulation in FL83B cells. C: cells incubated with the F12K medium containing the 100 nM insulin group. T: TNF-*α* induced for the insulin resistance group. OA: oleic acid induced for the fatty liver cells group. TA : TNF-*α* and oleic acid induced insulin resistance for the fatty liver cells group. ^*∗*^*p* < 0.05, ^*∗∗*^*p* < 0.01, and ^*∗∗∗*^*p* < 0.01 vs. control group; ^#^*p* < 0.05, ^##^*p* < 0.01, and ^###^*p* < 0.01 vs. TNF-*α* group; ^+^*p* < 0.05 and ^++^*p* < 0.01 vs. TA10 group; and ^$^*p* < 0.05 vs. TA120 group.

Both the OA-induced and the TNF-*α* plus OA-induced groups showed a significant increase in intracellular lipid accumulation (Figure 3). There was no difference between the groups administered EAf2 at 20, 30, and 60 min and the OA-induced group/TNF-*α* plus OA-induced group. However, a decrease in intracellular lipid accumulation was significantly observed 10 min after EAf2 administration, compared with the TA20 group (*p*=0.005), TA30 group (*p*=0.008), and TA60 group (*p*=0.012), respectively (Figure 4). After 120 min of EAf2 administration, the significantly decreased lipid accumulation was observed, compared with the TNF-*α*-induced IR group (*p*=0.045) (Figure 4).

### 3.4. Effect of EAf2 Fraction on TNF-*α* plus OA-Induced IR and Lipid Accumulation in Enhancing FL83B Cell Insulin Signaling Pathway Protein Expression

**Figure 5:**
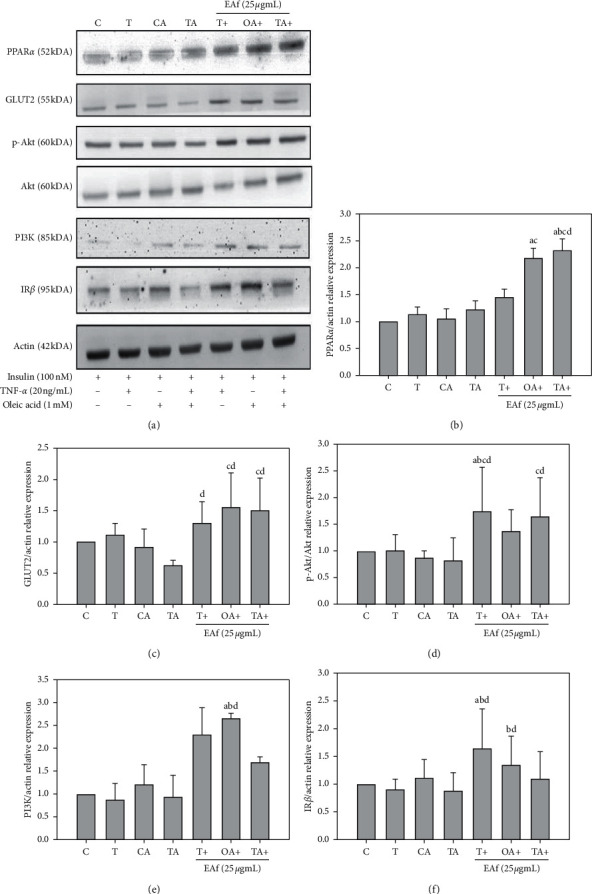
Effects of EAf2 on the expression of peroxisome proliferator-activated receptor alpha (PPAR*α*), glucose transporter-2 (GLUT2), phosphorylation of protein kinase B (PKB/Akt), phosphatidylinositol-3 kinase (PI3K), and insulin receptor-beta (IR*β*) in FL83B cells. C: cells incubated with the F12K medium containing the 100 nM insulin group. T TNF-*α* induced for the insulin resistance group. OA: oleic acid induced for the fatty liver cells group. TA : TNF-*α* and oleic acid induced insulin resistance for the fatty liver cells group. ^*a*^*p* < 0.05 vs. control group, ^*b*^*p* < 0.05 vs. TNF-*α* group, ^*c*^*p* < 0.05 vs. OA group, and ^*d*^*p* < 0.05 vs. TA group.

Western blot analyses of hepatic insulin-signaling-related proteins were conducted, including IR*β*, PI3K, p-Akt/Akt, GLUT2, and PPAR*α* (Figure 5(a)). PPAR*α* protein expression was increased after the EAf2 fraction administration to the OA-induced group and TNF-*α* plus OA-induced group, compared with the control group, which increased by 108% and 138%, respectively (Figure 5(b)). The expression of the GLUT2 protein increased after the administration of the EAf2 fraction to the TNF-*α*-induced, OA-induced, and TNF-*α* plus OA-induced groups (Figure 5(c)). The expression of the p-Akt/Akt protein increased after the administration of the EAf2 fraction to the TNF-*α*-induced and TNF-*α* plus OA-induced groups (Figure 5(d)). The administration of the EAf2 fraction increased PI3K protein expression in the OA-induced group (Figure 5(e)). The EAf2 fraction can regulate IR*β* protein expression in the TNF-*α*-induced and OA-induced groups (Figure 5(f)).

## 4. Discussion

**Figure 6:**
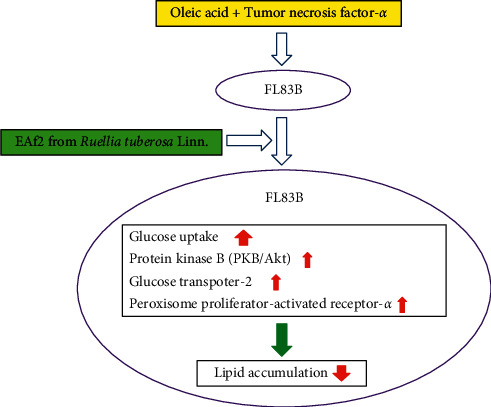
The postulated mechanism of EAf2 from RTL on improving glucose intake and inhibiting lipogenesis in FL83B mouse hepatocytes.

In the present study, EA fractions from RTL, especially in EAf2, showed the best improved glucose uptake in IR mouse FL83B hepatocytes. Additionally, the EAf2 fraction from RTL also attenuated IR plus lipid accumulation in FL83B cells. RTL may have possibly regulated and enhanced hepatic insulin-signaling-related metabolic protein expressions, including those of GLUT2, p-Akt/Akt, and PPAR*α* (Figure 6), suggesting that the possible underlying mechanisms of RTL extract were to improve glucose intake and inhibit lipogenesis in FL83B cells.

In this study, TNF-*α* inhibits glucose uptake in FL83B hepatocytes. OA increases lipid accumulation in TNF-*α*-treated FL83B hepatocytes. We postulated that the EAf2 fraction increased glucose uptake and suppressed lipid accumulation may be attributed by alleviating the IR in TNF-*α* and OA-treated FL83B hepatocytes. When adipocytes develop IR, insulin signaling is blocked. Lipogenesis is inhibited, lipolysis is increased, and FFA concentration is increased in the blood. The absorption rate of FFAs in other organs is increased, thereby stimulating hepatocytes for gluconeogenesis, fat synthesis, and synthesis of very-low-density lipoprotein (VLDL). No study has confirmed the lipotoxicity of liver fat and visceral fat in the IR of muscle cells, although obesity is associated with IR [12–14]. Individuals with higher liver and visceral fat who develop IR in muscle, liver, and fat cells show increased concentrations of lipoprotein in the blood, whereas individuals with obesity and higher visceral fat content have a significant increase in lipogenesis compared with individuals of normal weight [15]. The fat accumulated in liver cells reflects the balance of lipolysis, lipid peroxidation, lipogenesis, and VLDL production and elimination. When regulating fat metabolism dysfunction, hepatic fat decomposition is reduced, fat accumulation is increased, and fatty acid oxidation and VLDL production reach saturation, resulting in the accumulation of lipid in hepatocytes, which eventually leads to a fatty liver [4].

In the present study, IR*β*, PI3K, and p-Akt/Akt expressions were decreased in TNF-*α*-induced cells, compared with untreated cells, indicating that TNF-*α* induced IR in hepatocytes through this pathway. Moreover, TNF-*α* plus OA-induced cells were also altered through the same signaling pathway, which showed a decrease in the expression of both GLUT2 and PPAR*α*, illustrating that this model can induce IR in normal liver cells when combined with the fatty liver model.

Glucose uptake evaluation showed that the EAf2 fraction increased glucose uptake in IR FL83B cells, indicating that it can alleviate cellular IR and recover normal cellular insulin-related functions. The EAf2 fraction can enhance insulin receptor protein expression and stimulate PI3K expression, as well as promote the activation of downstream pathways. PI3K is a downstream molecule of the insulin receptor substrate (IRS), and when insulin binds to insulin receptors, IRS phosphorylation activation is promoted and the signaling of the PI3K pathway is activated. PI3K is a secondary signal transduction factor that subsequently promotes downstream Akt/PKB protein activation, and the glucose channel protein GLUT2 is moved from the cytoplasm to the surface of the cell membrane, thus enhancing cellular glucose uptake [16]. The EAf2 fraction has the effect of increasing intracellular phosphorylated Akt reaction, suggesting that the degree of phosphorylated Akt increases because of increased PI3K expression. When Akt is stimulated by upstream signaling, such as PI3K signaling, it activates Ser473 and Thr308 phosphorylation, which promotes downstream proteins, such as glycogen synthase kinase 3, forkhead box O, and mammalian target of rapamycin, to regulate glucose transport and glycogen synthesis [17]. The activation of Akt moves GLUT4 from the cytoplasm to the cell membrane, thereby increasing cellular glucose uptake. When insulin stimulation disappears, GLUT4 returns to the cytoplasm for standby. GLUT2 is glucose sensitive and has a bidirectional transport direction. It is present widely in the liver, islet beta cells, kidneys, and small intestine cells and is different from GLUT4. GLUT2 is regulated by insulin and glucose levels in the blood, which regulate the cellular glucose output or input [18].

IR combined with fatty liver results in reduced GLUT2 protein expression in liver cells. In the present study, the EAf2 fraction ameliorated IR combined with fatty liver by enhancing the expression of GLUT2 and PPAR*α*, thereby reducing intracellular lipid accumulation and ameliorating fatty liver. Indeed, the increased PPAR*α* protein expression reduced intracellular lipid accumulation [19]. PPAR*α* is abundant in the liver, muscle, and kidney. It is used to promote fat oxidation and remove FFAs in the blood, which has protective and preventive effects on fatty liver [19].

The administration of RTL fractions of n-hexane and EA in diabetic rabbits lowered the animals' blood sugar level [20]. Moreover, the best evaluation of antioxidant activity resulted from RTL extraction using methanol and EA [21]. Here, our findings showed that n-hexane and EA fractions of RTL have better effects on improving the glucose uptake capacity of IR cells, which is consistent with the results of other studies. EA fractions of RTL can effectively improve the blood sugar level in diabetes through their antioxidant capacity, as EA fractions speculatively contain high amounts of polyphenols and triterpenoids [20]. Another study indicated that the active compound of the EA fraction may be a polyphenol called cirsimaritin [22]. Although this compound is speculated, the components are quite complicated. Our recent study indicated the possible bioactive compounds of EA from RTL identified by high-performance liquid chromatography assay, including phenolic acids (syringic acid and p-coumaric acid) and flavanoid (cirsimaritin) on improving glucose uptake in hepatocytes [10].

## 5. Conclusions

Our results showed that EAf2 from RTL significantly increased glucose uptake and suppressed lipid accumulation in TNF-*α* plus OA-induced FL83B cells. The possible alleviating mechanism happens by enhancing the expression of the insulin signaling pathway protein, including Akt and GLUT2, to ameliorate the glucose uptake capacity, which may have been due to the increased Akt phosphorylation and GLUT2 expression that attenuated IR and the increased PPAR*α* expression that reduced lipid accumulation in TNF-*α* plus OA-induced FL83B cells. The further separation and identification of bioavailability contributor on alleviating abnormal lipid accumulation from RTL EA fractions using mouse hepatocyte models is currently on process in our laboratory.

## Data Availability

All the data used to support the findings of this study are included in the article.

## Supplementary Materials

Supplementary Materials Supplementary Figure 1. Thin-layer chromatography profiles of different column fractions from RTL. (A) Hf1: n-hexane layer fraction 1, (B) Hf2: n-hexane layer fraction 2, (C) Hf3: n-hexane layer fraction 3, (D) Hf4: n-hexane layer fraction 4, (E) EAf1: ethyl acetate fraction 1, (F) EAf2: ethyl acetate fraction 2, (G) EAf3: ethyl acetate fraction 3, and (H) EAf4: ethyl acetate fraction 4.

## References

[1] Boden G., She P., Mozzoli M. (2005). Free fatty acids produce insulin resistance and activate the proinflammatory nuclear factor- B pathway in rat liver. *Diabetes*.

[2] Samuel V. T., Shulman G. I. (2012). Mechanisms for insulin resistance: common threads and missing links. *Cell*.

[3] King G. L., Park K., Li Q. (2016). Selective insulin resistance and the development of cardiovascular diseases in diabetes: the 2015 edwin bierman award lecture. *Diabetes*.

[4] Gaggini M., Morelli M., Buzzigoli E., DeFronzo R., Bugianesi E., Gastaldelli A. (2013). Non-alcoholic fatty liver disease (NAFLD) and its connection with insulin resistance, dyslipidemia, atherosclerosis and coronary heart disease. *Nutrients*.

[5] Mu W., Cheng X.-f., Liu Y. (2019). Potential nexus of non-alcoholic fatty liver disease and type 2 diabetes mellitus: insulin resistance between hepatic and peripheral tissues. *Frontiers in Pharmacology*.

[6] Cui W., Chen S. L., Hu K. Q. (2010). Quantification and mechanisms of oleic acid-induced steatosis in HepG2 cells. *American Journal of Translational Research*.

[7] Zhang D.-D., Zhang J.-G., Wu X. (2015). Nuciferine downregulates Per-Arnt-Sim kinase expression during its alleviation of lipogenesis and inflammation on oleic acid-induced hepatic steatosis in HepG2 cells. *Frontiers in Pharmacology*.

[8] Ko C.-Y., Lin R.-H., Zeng Y.-M. (2018). Ameliorative effect of *Ruellia tuberosa L.* on hyperglycemia in type 2 diabetes mellitus and glucose uptake in mouse C2C12 myoblasts. *Food Science & Nutrition*.

[9] Chang W.-C., Huang D.-W., Chen J.-A., Chang Y.-F., Swi-Bea Wu J., Shen S.-C. (2018). Protective effect of *Ruellia tuberosa* L. extracts against abnormal expression of hepatic detoxification enzymes in diabetic rats. *RSC Advances*.

[10] Xu J. H., Lo Y. M., Chang W. C. (2020). Identification of bioactive components from *Ruellia tuberosa* L. on improving glucose uptake in TNF-*α*-induced insulin-resistant mouse FL83B hepatocytes. *Evidence-Based Complementary and Alternative Medicine*.

[11] Shen S.-C., Chang W.-C., Chang C.-L. (2013). An extract from wax apple (Syzygium Samarangense (blume) merrill and perry) effects glycogenesis and glycolysis pathways in tumor necrosis factor-*α*-treated fl83b mouse hepatocytes. *Nutrients*.

[12] Bugianesi E., Gastaldelli A., Vanni E. (2005). Insulin resistance in non-diabetic patients with non-alcoholic fatty liver disease: sites and mechanisms. *Diabetologia*.

[13] Gastaldelli A., Cusi K., Pettiti M. (2007). Relationship between hepatic/visceral fat and hepatic insulin resistance in nondiabetic and type 2 diabetic subjects. *Gastroenterology*.

[14] Kotronen A., Juurinen L., Hakkarainen A. (2008). Liver fat is increased in type 2 diabetic patients and underestimated by serum alanine aminotransferase compared with equally obese nondiabetic subjects. *Diabetes Care*.

[15] Wajchenberg B. L. (2000). Subcutaneous and visceral adipose tissue: their relation to the metabolic syndrome. *Endocrine Reviews*.

[16] Czech M. P., Corvera S. (1999). Signaling mechanisms that regulate glucose transport. *Journal of Biological Chemistry*.

[17] Huang X., Liu G., Guo J., Su Z. (2018). The PI3K/AKT pathway in obesity and type 2 diabetes. *International Journal of Biological Sciences*.

[18] Leturque A., Brot-Laroche E., Le Gall M. (2009). GLUT2 mutations, translocation, and receptor function in diet sugar managing. *American Journal of Physiology. Endocrinology and Metabolism*.

[19] Chang J.-J., Hsu M.-J., Huang H.-P., Chung D.-J., Chang Y.-C., Wang C.-J. (2013). Mulberry anthocyanins inhibit oleic acid induced lipid accumulation by reduction of lipogenesis and promotion of hepatic lipid clearance. *Journal of Agricultural and Food Chemistry*.

[20] Shahwara D., Ullah S., Ahmad M., Ullah S., Ahmad N., Khan M. A. (2011). Hypoglycemic activity of *Ruellia tuberosa* linn (Acanthaceae) in normal and alloxan-induced diabetic rabbits. *Indian Journal of Pharmaceutical Sciences*.

[21] Chen F.-A., Wu A.-B., Shieh P., Kuo D.-H., Hsieh C.-Y. (2006). Evaluation of the antioxidant activity of *Ruellia tuberosa*. *Food Chemistry*.

[22] Wulan D. R., Utomo E. P., Mahdi C. (2015). Antidiabetic activity of Ruellia tuberosa L., role of *α*-amylase inhibitor: in silico, in vitro, and in vivo approaches. *Biochemistry Research International*.

