# Supplementary material for: Alleviative Effect of Ruellia tuberosa L. on Insulin Resistance and Abnormal Lipid Accumulation in TNF-α-Treated FL83B Mouse Hepatocytes
Source: Evid Based Complement Alternat Med. 2021 Jun 23;2021:9967910. doi: 10.1155/2021/9967910 (PMC8249146; doi:10.1155/2021/9967910)
Supplement: Supplementary Materials — Supplementary Figure 1. Thin-layer chromatography profiles of different column fractions from RTL. (A) Hf1: n-hexane layer fraction 1, (B) Hf2: n-hexane layer fraction 2, (C) Hf3: n-hexane layer fraction 3, (D) Hf4: n-hexane layer fraction 4, (E) EAf1: ethyl acetate fraction 1, (F) EAf2: ethyl acetate fraction 2, (G) EAf3: ethyl acetate fraction 3, and (H) EAf4: ethyl acetate fraction 4. [file 9967910.f1.docx]

**Supplementary Figure 1**


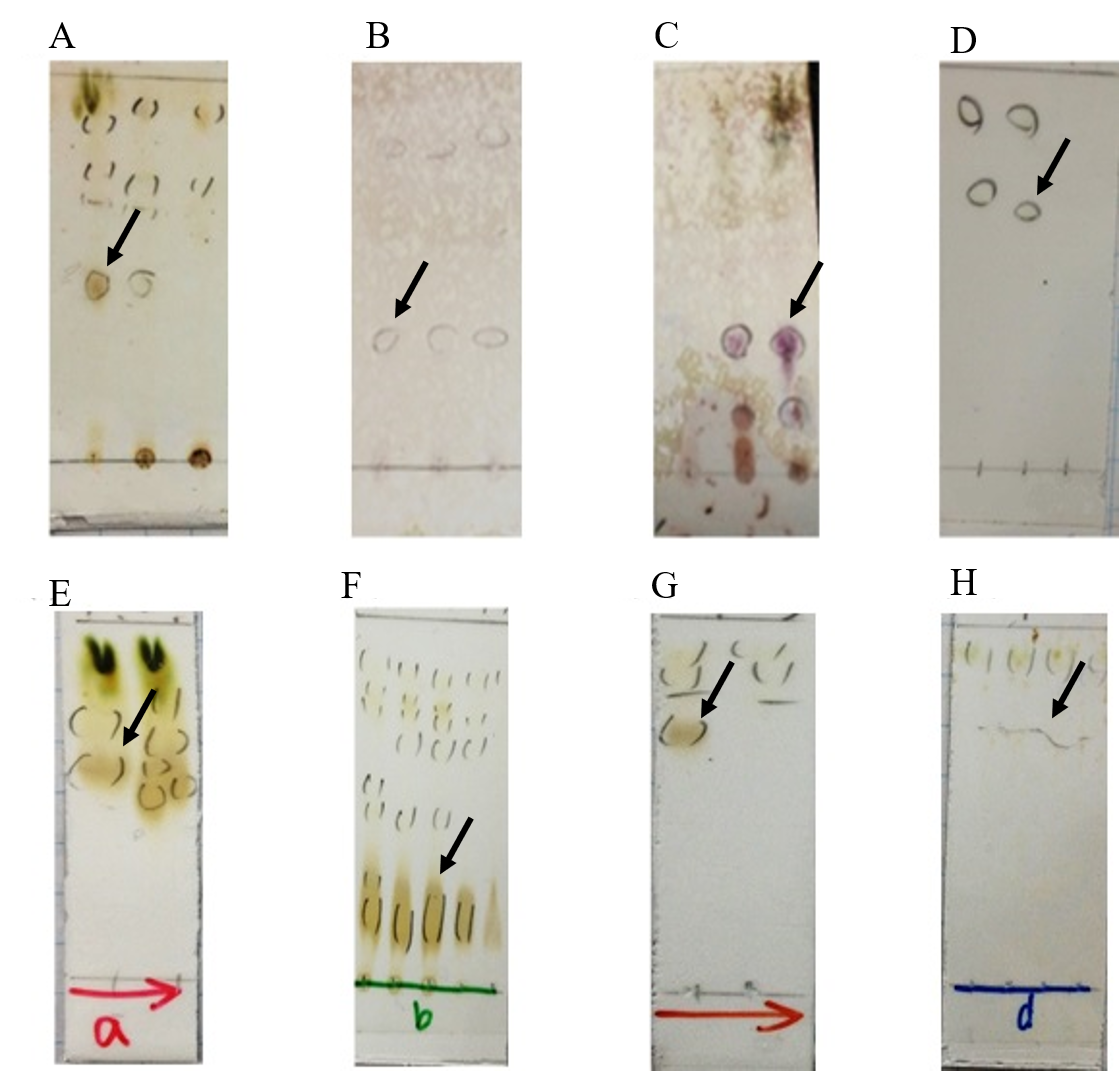


**Supplementary Figure 1. Thin layer chromatography profiles of different column fractions from RTL.** (A) Hf1: n-hexane layer fraction 1, (B) Hf2: n-hexane layer fraction 2, (C) Hf3: n-hexane layer fraction 3, (D) Hf4: n-hexane layer fraction 4, (E) EAf1: ethyl acetate fraction 1, (F) EAf2: ethyl acetate fraction 2, (G) EAf3: ethyl acetate fraction 3, (H) EAf4: ethyl acetate fraction 4.
